# The Role of Cognitive Emotion Regulation for Making and Keeping Friend and Conflict Networks

**DOI:** 10.3389/fpsyg.2022.802629

**Published:** 2022-04-25

**Authors:** Courtney Ricciardi, Olga Kornienko, Pamela W. Garner

**Affiliations:** ^1^Department of Psychology, George Mason University, Fairfax, VA, United States; ^2^School of Integrative Studies, George Mason University, Fairfax, VA, United States

**Keywords:** emotion regulation, social network analysis, stochastic actor-oriented model, friendship, conflict

## Abstract

We used social network analysis (SNA) to examine how adaptive ER strategies (acceptance, positive reappraisal, refocusing, and putting in perspective) and maladaptive ER strategies (rumination, catastrophizing, self- and other-blame) predict the creation and maintenance of friendship and conflict relationships within a mixed-gender social group. Participants (*n* = 193, 53% female, *M* age = 19.4 years, 62.1% White) reported on emotion regulation, friendship, and conflict nominations at two time points. Stochastic actor-oriented models revealed that similarity in endorsement of adaptive ER strategies predicted maintenance of friendship and conflict relationships over time. However, new conflict relationships were more likely to form between those who differed in use of adaptive ER. Finally, more frequent use of maladaptive ER strategies was related to termination of existing conflict ties and the creation of new ones. Deploying social network analysis as a methodology for examining social relationships enables the unpacking the dynamics of multiple social relationships (friend and conflict), identifying the role of ER for structuring of social relationships among group members. Although cognitive ER is an intra-individual process, it fundamentally occurs within a social environment and our results advance the knowledge of how ER contributes to how this social environment is created in a first place.

## Introduction

It is well-established that the adaptive regulation of emotion enhances the quality and longevity of social relationships (Lopes et al., 2011; English et al., 2012; Garner and Waajid, 2012) and contributes to short- and long-term well-being across development (Djambazova-Popordanoska, 2016; Chervonsky and Hunt, 2019; Cloitre et al., 2019). Still, scholars have called for more research centered on understanding associations among ER, interpersonal mechanisms, group dynamics, and other social consequences of emotions (e.g., Zaki and Williams, 2013; English and Eldesouky, 2020). These calls converge on the proposition that ER is influenced by the social context and vice versa. Although much less effort has been directed at uncovering the role of ER in shaping relationships within social groups. A notable exception is socioemotional selectivity research, which has shown that the selective narrowing of social networks as a function of aging improves emotional experiences and may operate as an antecedent ER strategy (English and Carstensen, 2014). Drawing upon these ideas, the current study uses social network analysis (SNA) to examine how the use of self-reported cognitive ER strategies contributes to young adults’ ability to make new and keep existing friendships and conflict-laden relationships, a possibility that has been underappreciated in individual-oriented psychological research.

Regulating one’s emotions requires a consideration of how emotions are elicited and appraised and involves the application of behavioral and cognitive strategies that operate to change the emotion and/or its expression (Bargh and Williams, 2007; Gross and Thompson, 2007). The focus in this research is on *cognitive* ER strategies, which can be categorized as adaptive or maladaptive when one is coping with a stressful event (Garnefski and Kraaij, 2006). Cognitive reappraisal and acceptance are regarded as *adaptive* ER strategies because they effectively de-escalate negative emotions (Gross and John, 2003; Mauss et al., 2007). High regulatory competence predicts increased popularity as a friend (Lopes et al., 2005) and specific ER strategies, such as cognitive reappraisal and co-rumination (at least for girls), have been linked to enhanced friendship quality (Gross and John, 2003; Felton et al., 2019), the downregulation of anger and other negative emotions (Mauss et al., 2007; Lennarz et al., 2019), and the upregulation of positive affect (Gross and John, 2003). Conversely, self-blame, and catastrophizing are considered *maladaptive* cognitive ER strategies because they prolong and exacerbate anger, stress, depression, and anxiety (Naragon-Gainey et al., 2017; Garnefski and Kraaij, 2018). Maladaptive ER strategies also forecast challenges in social functioning and can perpetuate negative social relationships (e.g., Mihalca and Tarnavska, 2013; von Salisch and Zeman, 2018). Moreover, the inability to manage negative affect is associated with dissatisfying friendships (Berry et al., 2000; Campos et al., 2015).

Importantly, positive and negative social relationships are interconnected, such that focusing exclusively on one or the other may distort the understanding of the relational dynamics that occur within social groups and underestimate the role of ER strategies in social network dynamics (Harrigan et al., 2020). Although positive reappraisal and refocusing are essential elements of friendship development and maintenance (Demır and Weitekamp, 2007), researchers know very little about the role of ER strategy use in how negative relationships form and change over time. Negative social relationships embody negative emotions and goal impairment (Wiseman and Duck, 1995). The use of maladaptive ER strategies, such as rumination and suppression has been linked to elevated conflict levels in social relationships (Miller et al., 2004; Kim et al., 2009; Webb et al., 2012). In contrast, the use of maladaptive ER strategies exacerbate conflict over time (Shulman et al., 2006; Lopes et al., 2011). Likewise, when relationship partners are low in the use of adaptive ER strategies, they are especially likely to attribute hostility to feedback from others (Klein et al., 2016). Longitudinal links among friendship, conflict, and problematic social problem-solving strategies also have been documented, but only for youth with low ER ability (Clayton et al., 2019). A priority of the current study was to distinguish between contribution of ER strategy use to different types of relationships (i.e., friendship and conflict) and phases of relationship development such as formation and maintenance (Neal and Veenstra, 2021).

### Unpacking Multidimensionality and Dynamics Within Social Networks

Understanding the dynamic processes through which ER strategies contribute to the creation and maintenance of social *networks* requires adopting the lens of network science and using SNA methods. Broadly speaking, networks represent building blocks of group living that evolve over time and are guided by multiple social processes that include ER strategies. Understanding how ER strategies predict the creation and maintenance of positive and negative relationships requires: a) a focus on *who is* and *who is not* chosen as a friend, b) an account of *who is* and *who is not* experiencing a conflict-laden relationship, and c) attention to the multiple selection processes through which theses social connections are formed (Snijders et al., 2010). SNA allows understanding multiple processes through which an individual attribute, such as ER, can be associated with social network dynamics. These processes include (1) *network activity*, which assesses how ER is associated with a tendency of the focal individual to send out connections; (2) *network popularity*, which measures how ER is associated with a tendency of a focal individual to receive incoming nominations from other group members; and (3) *homophily*, which describes the tendency to befriend similar others (Snijders et al., 2010). These distinctions may be especially important for determining how ER strategy use influences relationship formation because ER affects both how one views their relationships *and* how one is perceived by others.

Finally, social networks evolve through multiple, intertwined processes, which are collectively referred to as network selection processes. Providing an unbiased account of how ER contributes to network selection requires statistically controlling for other co-occurring selection processes (Snijders et al., 2010). Network selection processes include two broad categories: (a) those based on individual characteristics (e.g., sex and age; McPherson et al., 2001) and (b) those reflecting how connections between individuals depend on their connections with other members of a group (e.g., whether two people have a friend in common; Rivera et al., 2010). Because multiple processes operate jointly in producing network structure, inferring which process is responsible for observed networks is challenging. To characterize the role of an attribute, such as ER, for network selection, one must first statistically control for co-occurring and, thus, confounding network processes using advanced SNA methods (Snijders et al., 2010). Failure to account for these alternative processes likely leads to an overestimation of contributions of ER to network selection.

### Linking Emotion Regulation to Making and Keeping Friendship Networks

Although no research to date has examined how cognitive ER strategy use is associated with making new friends and keeping existing ones in a social network paradigm, evidence from individual and dyadic levels of analysis is instructive. Competence in regulating one’s emotions is associated with increased peer popularity, having a higher number of mutual friends, and can expand one’s friendship ties (Philippot et al., 2004). Considering specific ER strategies, cognitive reappraisal is positively linked with favorable affective displays and the quality of social relationships (Gross and John, 2003). In contrast, the use of maladaptive ER strategies, such as withdrawal, aggressive behavior, and giving up, is associated with a greater likelihood that individuals will experience difficulty forming and sustaining friendships (Reindl et al., 2016).

These results suggest that individuals with higher levels of adaptive ER strategies would be more popular as potential friends and, as such, they would receive more new and keep more existing friendship nominations. Individuals endorsing maladaptive ER strategies may be less likely to initiate new and maintain existing incoming friendships and more likely to be avoided by peers because of increased negative emotions displays of aversive interaction patterns (Butler et al., 2003; Reindl et al., 2016). A similar pattern of association also is plausible for the association between maladaptive ER strategies and the number of outgoing friendships.

The final mechanism through which ER strategies could shape friendship connections is homophily. Social networks are formed based on similar socio-demographic characteristics and behavior (McPherson et al., 2001), including happiness (van Workum et al., 2013). Individuals who endorse adaptive ER strategies may prefer to befriend others who also display successful coping strategies. As well, those who endorse maladaptive ER strategies may show a preference for befriending individuals that share their same level of undesirable ER strategy use, as suggested by patterns of co-rumination between friends (Byrd-Craven et al., 2011). Preferences for similarity may be especially pronounced for the maintenance of existing friendships because similarity promotes ease of communication and reduce conflict (McPherson et al., 2001).

### Linking Emotion Regulation to Making and Keeping Conflict Networks

In the absence of prior studies linking cognitive ER strategies to conflict network dynamics, we draw on a smaller body of evidence that generally suggests that adaptive ER strategies are inversely linked to making and retaining conflict-laden relationships (Shulman et al., 2006; Lopes et al., 2011). Similarly, studies of marital satisfaction report that faster downregulation of negative emotion is related to marital satisfaction (Bloch et al., 2014), suggesting that ER strategies, which extend the experience of negative emotion may lead to a decrease in relational satisfaction. Additionally, when relationship partners are low in the use of adaptive ER strategies, they are especially likely to perceive one another’s feedback as hostile (Klein et al., 2016), which could create and perpetuate conflict. Just as friendships exist in networks, and are affected by the ways in which individuals regulate their negative emotions, conflict-laden relationships also can emerge among group members and be shaped by individual attributes. Individuals with a tendency to implement adaptive ER strategies may receive fewer incoming conflict ties based on their increased ability to manage distress (i.e., decreased popularity in a conflict network). Further, individuals who use maladaptive ER strategies may be particularly likely to accrue high numbers of outgoing conflict ties due to their inability to cope constructively with negative emotions.

### Present Study

Relying upon evidence that cognitive ER strategies impact the quality and quantity of relationships, we investigate whether high endorsement of adaptive cognitive ER strategies predict high rates of friendship creation and maintenance over time. Within friendship network dynamics, it is possible that individuals who use adaptive ER will create and maintain more incoming and outgoing ties and seek out friends who employ similar levels of adaptive ER strategies. The opposite pattern of associations between maladaptive ER and network dynamics appears plausible, but, in the absence of prior research, these analyses were exploratory in nature. Similarly, our analyses of the role of ER strategies in conflict network creation and termination remain exploratory given the scarcity of past research.

**FIGURE 1:**
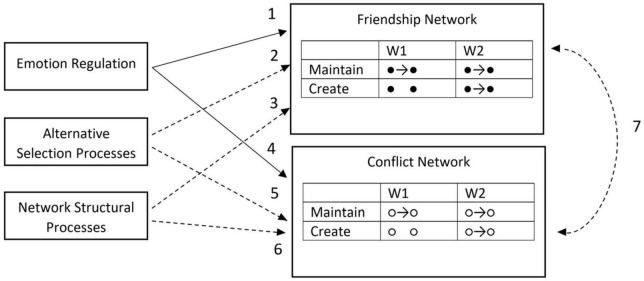
Conceptual diagram of how emotion regulation contributes to friendship and conflict network selection dynamics in social groups. Paths are numbered in the order they are discussed in the text. Solid lines represent the main paths of interest examined in this study; dashed lines represent paths describing alternative social network selection processes that we statiscally account for by simulataneous inclusion in our model.

We use stochastic actor-oriented modeling (SAOM; Snijders et al., 2010) to investigate the extent to which ER strategies predict friendship and conflict network dynamics, while accounting for a host of alternative processes that also give rise to networks (see Figure 1 for a graphic overview). Our primary interest is in understanding how adaptive and maladaptive ER predict relationship dynamics (paths 1 and 4), but we also account for confounding social processes, such as alternative selection on socio-demographic (i.e., gender, ethnicity, and race; McPherson et al., 2001) and band-related characteristics (i.e., band section) in both types of networks (paths 2 and 5). Finally, we include model parameters to account for how network structural effects explain the evolution of the friendship and conflict-laden networks (paths 3 and 6; Rivera et al., 2010) as well as cross-network effects (path 7). Controlling for these confounding processes and joint estimation of selection effects produces less biased estimates of associations between ER strategy use and network dynamics than individual-level inferential statistics.

## Method

### Participants and Setting

Participants from this study include members of a large, collegiate marching band in the Southwestern United States. Consent was obtained from 72% of band members (*N* = 220) and 63% of band members completed all aspects of the study (*N* = 193). This sample was balanced in gender (53% female); and composed of 63.7% White, 19.7% Latino, 5.2% Black, 5.2% Asian, 3.6% Native American, and 2.1% “Other.” The average age of participants was 19.44 years (*SD* = 1.51) and participants had completed anywhere from 1 to 6 seasons (*M* = 2.17, *SD* = 1.19). The band is broken down into sections organized by instrument. Sections included anywhere from 11 to 28 people.

### Procedures

In the fall semester, participants were given log-in credentials that allowed them to take a 30-min online survey that included topic areas such as emotion regulation personality, perceived stress, and demographic information. The survey was completed 1 week prior to an in-person assessment in which network measures including a peer nominations inventory were collected. There were two time data collections during the semester, the first in September and the second in November. All procedures were approved by the university’s Office of Research Integrity and Assurance.

### Measures

#### Demographic Measures

At time one, participants responded to a survey about their demographic information, specifically reporting their age, gender, ethnicity, and prior experience in college-level marching.

#### Emotion Regulation Strategy Use

ER regulation strategy use was measured with the Cognitive Emotion Regulation Questionnaire (CERQ), short version (Garnefski and Kraaij, 2006). Four adaptive ER strategies were included: putting in perspective (e.g., “I think it could have all been much worse”), acceptance (e.g., “I think I cannot change anything about it”), positive reappraisal (e.g., “I think I can learn something from the situation”) and refocusing (e.g., “I think of something nice instead of that has happened”), and four maladaptive ER strategies; rumination (e.g., “I dwell upon the feelings the situation has evoked in me”), catastrophizing (e.g., “I continually think about how horrible the situation has been”), self-, and other blame (e.g., “I think that basically the cause must lie within myself” and “I feel that others are responsible for what has happened”, respectively). Cronbach alphas were all above 0.72. Participants responded to four items for each subscale on a scale of 1–5 where “1” indicated participating in the included behavior *almost never*, and “5” indicated participating in the included behavior *almost always*. A summary score for each subscale was obtained and scores for perspective-taking, acceptance, positive reappraisal, and refocusing were composited to create the adaptive ER strategy variable. The composite score for maladaptive ER strategy use was an aggregate of rumination, catastrophizing, and self- and other-blame.

#### Social Networks

Participants were given a list of names and ID codes for every participating member of the band and instructed to “list the ID codes of band-mates who are your closest friends with whom you spend a lot of time doing different activities and whom you can count on when you need help.” Participants were also instructed to list the ID codes of those with whom they have a conflict, “individuals with whom you had experienced interpersonal conflict, tension, or with whom you just did not get along.” There was no limit on the number of people participants could nominate at two time points in the semester. Nominations were organized in a binary matrix, where a directed friendship or conflict tie (i.e., person A nominated person B) was coded as 1.

### Analytical Strategy

Stochastic actor-oriented modeling was used to estimate the effect of ER strategy use on friendship and conflict creation and maintenance over time. SAOMs makes several assumptions: a) network ties are enduring states rather than temporary events, b) individuals in the network control their ties and change them one at a time, which leads to a cascading effect of changes within the network, and c) each actor has full knowledge of the network and all of its actors (Snijders et al., 2010). Our data are in line with these assumptions. Interested readers are directed to comprehensive reviews of statistical modeling of social networks by Snijders et al. (2010), Snijders (2011), and Robins (2013, 2015).

### Model Effects

We included a number of network structural effects including outdegree, or how common friendship was overall in the network, reciprocity, how often friendship was mutual in the network, and transitivity. To measure transitivity, or triadic dependence, we included transitive triplets which measures the tendency for friends of friends to be friends, transitive reciprocated triplets which interacts reciprocity and transitivity, transitive ties to estimate the effect of having an intermediary tie on tie formation, 3-cycles to estimate the effect of having multiple intermediary ties on tie formation, balance which measures the tendency for ties to be structurally similar, and the number of actors at distance 2 or the preference for keeping others at a social distance of 2. Indegree and outdegree popularity, along with outdegree activity were included in order to model variance and covariance of in and outdegrees. More specifically, indegree popularity estimates were more likely to develop additional ties over time. The square root form of each of these was used.

The model also included a number of effects for selection on demographic covariates. Homophily was included for race, gender, age, section, section leader status, and seasons. These effects measured whether similarity on the relevant trait was related to an increase in the likelihood of friendship ties. For example, did females express a preference for friendship with other females. Ego effects were included for band section leader status and seasons, and were used to measure how being a section leader and number of seasons in band were related to social activity. Alter effects were also included for section leader and seasons to measure whether these were related to popularity. The conflict model also included outdegree density, reciprocity, and popularity and activity to model network structural effects and similar covariates.

To directly address the research questions, we included ego, alter, and homophily effects for adaptive and maladaptive ER strategy use on tie maintenance and creation in both the friendship and conflict-laden networks. Maintenance and creation were considered separately. Ego effects for ER on friendship creation measures how higher endorsement of adaptive or maladaptive ER tendencies is related to new outgoing ties over time. Alter effects for ER strategy use on friendship creation measures how higher endorsement of adaptive or maladaptive ER is related to new incoming ties over time. Finally, homophily effects for ER strategy use on friendship formation measure how similarity in ER strategy use is related to creating new friendships. The same pattern applies to friendship and conflict maintenance, such that ego effects illustrate the association between ER strategy use and the actor to retain friendships or conflict ties, alter effects denote how ER strategy use relates to others tendency to maintain friendships or conflicts with actor, and homophily effects demonstrate how similar endorsement of ER use related to both actors remaining in a friendship of conflict relationship.

## Results

Descriptive statistics regarding ER strategy use, friendship and conflict networks are presented in Table 1. Among 193 network members, there were the total of 1,204 friendship ties at time one and 1,117 friendship ties at time two. At both timepoints, at the level of the system, approximately 3% of possible connections existed, as evidenced by global network density estimates. This type of sparsely connected network is typically observed for friendship relationships. On average, each individual nominated 6.238 friends at time one and 5.788 friends at time two. The Jaccard index of 0.321 suggested that 32% of friendship ties present at one time point were present at both points. This can be used to interpret stability within the network and meets conventional thresholds for stability in SAOM modeling (Snijders et al., 2010). The conflict network had the total of 241 ties at time 1 and 285 ties at time 2. Less than 1% of possible conflict ties were observed at the network level as indicated by network density. On average, each individual nominated 1.249 individuals with whom they had conflict at time 1 and 1.477 individuals at time 2. The Jaccard index of 0.235 suggested that 23.5% of conflict ties present at one time point were present at both points.

**TABLE 1:** Descriptive statistics.

Descriptive statistics		
	**Time 1**	**Time 2**
**Adaptive ER**		
Mean (SD)	12.79 (2.72)	
Range	7.00–19.50	
**Maladaptive ER**		
Mean (SD)	10.05 (2.44)	
Range	5.00–18.75	
**Friendship network**		
Outdegree	6.238	5.788
Density	0.032	0.03
Number of ties	1,204	1,117
Jaccard index	0.321	
**Conflict network**		
Outdegree	1.249	1.477
Density	0.007	0.008
Number of ties	241	285
Jaccard index	0.235	

### Predicting Friendship Network Dynamics

A SAOM was used to analyze how the use of adaptive and maladaptive ER strategies were associated with changes in friendship and conflict networks (Table 2). Results revealed that those with similar levels of adaptive ER strategy use were more likely to maintain friendships over time (est. = 1.07, *p* < 0.05; path 1 in Figure 1). Adaptive ER strategy use was not related to the likelihood of friendship nomination, nor to the number of outgoing friendship ties extended. Endorsement of maladaptive ER strategy use was not associated with friendship network ties.

**TABLE 2:** Stochastic actor based model results for contributions of emotion regulation to selection in friendship and conflict networks.

Effects	Estimate	S.E.	p
**Emotion regulation and friendship selection (path 1 in Figure 1)**			
**Maintenance**			
Adaptive ER alter	–0.02	(0.03)	
Adaptive ER ego	–0.53	(0.47)	
Adaptive ER similarity	1.07	(0.51)	*
Maladaptive ER alter	0.04	(0.03)	
Maladaptive ER ego	–0.50	(0.62)	
Maladaptive ER similarity	0.30	(0.67)	
**Creation**			
Adaptive ER alter	–0.02	(0.03)	
Adaptive ER ego	0.47	(0.47)	
Adaptive ER similarity	–0.56	(0.54)	
Maladaptive ER alter	0.01	(0.03)	
Maladaptive ER ego	0.46	(0.62)	
Maladaptive ER similarity	–0.18	(0.75)	
**Alternative friendship selection processes (path 2)**			
Same female	–0.09	(0.07)	
Same race	0.16	(0.07)	*
Same section	0.56	(0.08)	*
Band section leader alter	–0.03	(0.13)	
Band section leader ego	–0.33	(0.13)	*
Band section leader similarity	0.02	(0.12)	
Seasons alter	0.07	(0.04)	
Seasons ego	–0.03	(0.04)	
Seasons similarity	0.43	(0.20)	*
**Friendship network structural effects (path 3)**			
Rate	12.93	(0.87)	
Outdegree (density)	–2.76	(1.18)	*
Reciprocity	1.95	(0.17)	*
Transitive triplets	0.17	(0.09)	
Transitive reciprocated triplets	–0.20	(0.07)	*
3-cycles	0.04	(0.08)	
Transitive ties	0.46	(0.12)	*
Balance	0.00	(0.04)	
Number of actors at dist 2	–0.11	(0.05)	*
Indegree–popularity (sqrt)	0.26	(0.07)	*
Outdegree–popularity (sqrt)	–0.18	(0.33)	
Outdegree–activity (sqrt)	–0.01	(0.26)	
**Emotion regulation and conflict selection (path 4)**			
**Maintenance**			
Adaptive ER alter	–0.10	(0.08)	
Adaptive ER ego	–0.39	(0.33)	
Adaptive ER similarity	2.72	(1.29)	*
Maladaptive ER alter	–0.14	(0.11)	
Maladaptive ER ego	–1.08	(0.37)	*
Maladaptive ER similarity	–2.57	(2.16)	
**Creation**			
Adaptive ER alter	–0.03	(0.04)	
Adaptive ER ego	0.21	(0.27)	
Adaptive ER similarity	–1.44	(0.66)	*
Maladaptive ER alter	–0.04	(0.05)	
Maladaptive ER ego	0.71	(0.32)	*
Maladaptive ER similarity	–1.33	(1.14)	
**Alternative conflict selection processes (path 5)**			
Same race	–0.05	(0.19)	
Same section	1.99	(0.22)	*
Band section leader alter	0.55	(0.27)	*
Band section leader ego	–0.12	(0.30)	
Band section leader similarity	0.32	(0.26)	
Seasons alter	–0.05	(0.10)	
Seasons ego	0.18	(0.09)	
Seasons similarity	1.12	(0.47)	*
**Conflict network structural effects (path 6)**			
Rate	4.08	(0.48)	
Outdegree (density)	–6.66	(0.58)	*
Reciprocity	0.89	(0.33)	*
Indegree–popularity (sqrt)	0.30	(0.16)	
Outdegree–popularity (sqrt)	0.74	(0.29)	*
Outdegree–activity (sqrt)	0.60	(0.16)	*
**Cross-network effects (path 7)**			
Conflict to agreement effect in friend network	–0.09	(0.17)	
Friend to agreement effect in conflict network	0.70	(0.15)	*

*ER, emotion regulation.*

*Convergence t-ratios for each parameter was less than 0.1. Overall maximum convergence ratio 0.12.*

*p < 0.05. Goodness of fit analyses are presented in Supplementary Material.

Associations between ER strategy use and friendship network processes were documented while accounting for several confounding network selection processes (path 2), which we consider next. Friendships were more likely between those of the same race (est. = 0.16, *p* < 0.05), in the same band section (est. = 0.56, *p* < 0.05), and among those who had a similar tenure in the band (seasons; est. = 0.43, *p* < 0.05). Band section leaders extended fewer friendship nominations than regular band members (est. = −0.33, *p* < 0.05). Gender was not associated with friendship. As expected, most structural network processes emerged in the friendship network (path 3). The following network structural effects were also included to model endogenous processes through which friendship network was selected. The high degree of reciprocity (est. = 1.95, *p* < 0.05) observed suggested that a high degree of reciprocal friendship nominations. The transitive reciprocated triplets parameter was negative (est. = −0.20, *p* < 0.05), which can be understood to mean that either transitivity or reciprocity could support friendship selection. Similarly, the negative parameter representing the number of actors at distance 2 (est. = −0.11, *p* < 0.05) suggested that band members were not friends unless they have friends in common. Finally, the positive indegree popularity parameter (est. = 0.26, *p* < 0.05) suggested that those who received a high number of incoming friendship ties at time one were more likely to receive additional friendship ties at time two (i.e., popularity begets popularity).

### Predicting Conflict Network Dynamics

Within the conflict network, we see that similar endorsement of adaptive ER was related to an increase in the likelihood of maintaining conflicts over time (est. = 2.72, *p* < 0.05; path 4). Contemporaneously, new conflicts were less likely to form between those who endorsed similar levels of adaptive ER (est. = −0.39, *p* < 0.05). Adaptive ER strategy use was not associated with the number of outgoing or incoming conflict ties over time. The negative parameter for ER ego maintenance suggests that those who reported higher use of maladaptive ER strategies were less likely to maintain conflicts over time, or in essence, those who endorsed higher maladaptive ER were more likely to terminate conflicts over time (est. = −1.08, *p* < 0.05). However, those who endorsed higher levels of maladaptive ER were also more likely to create new conflicts over time (est. = 0.71, *p* < 0.05). The employment of maladaptive ER was not related to the maintenance or creation of incoming conflict ties, nor was similarity in levels of maladaptive ER associated with the likelihood of conflict between band members.

These effects in conflict network emerged when controlling for other conflict network selection (path 5) and network structural (path 6) effects. Conflicts were more likely between those in the same band section (est. = 1.99, *p* < 0.05) and among those who had been in the band for roughly the same amount of time (est. = 1.12, *p* < 0.05). Band section leaders received more conflict nominations than other members of the band (est. = 0.55, *p* < 0.05). Race was not associated with the likelihood of conflicted relationships. The following network structural effects were documented: outdegree popularity parameter (est. = 0.74, *p* < 0.05) and the outdegree activity parameter (est. = 0.60, *p* < 0.05) were significant suggesting that those who extended more conflict ties at time one both send out and receive more conflict ties at time two.

### Cross-Network Effects

Finally, because we estimated friendship and conflict network selection at the same time, we were able to account for cross-network associations between the two networks, or examine how existence of friendships was associated with existence of conflict relationships (path 7). Our findings indicated evidence for an interpersonal process that could be described as “the enemies of my friends are my enemies” (friend to agreement effect in conflict network; est. = 0.70, *p* < 0.05). This suggests that band members established conflict-laden relationships with others with whom their friends previously had conflict.

### Goodness of Fit

Following standard procedures for assessing goodness of fit, model-implied simulated networks were compared to the observed data for key network properties (Hunter et al., 2008; Ripley et al., 2022). Fit was assessed for outdegree, indegree, geodesic distances, and triad census for both friendship and conflict networks (Lospinoso et al., 2011). This was done through the sienaGOF function which compares observed values to simulated values and asses that difference using Mahalanobis distance. When this difference is *p* > 0.05, the predicted distribution does not differ from the observed significantly, thus indicating adequate fit. GOF results from the friendship model (available in the Supplementary Material) show that the distribution of outdegrees (*p* = 0.36), indegrees (*p* = 0.26), geodesic distances (*p* = 0.58), and triadic configurations (*p* = 0.66) in the model-implied simulated networks were not significantly different from the distribution for these configurations in the observed network. GOF results from the conflict model (available in the Supplementary Material) show that the distribution of outdegrees (*p* = 0.18), indegrees (*p* = 0.41), geodesic distances (*p* = 0.68), and triadic configurations (*p* = 0.14) in the model-implied simulated networks were not significantly different from the distribution for these configurations in the observed conflict network. Thus, we observed good to excellent model fit for the friendship and conflict networks.

## Discussion

Recently, there has been an increased number of calls to understand interpersonal mechanisms, group dynamics, and social consequences of ER (e.g., English and Eldesouky, 2020). These proposals converge on the idea that ER fundamentally occurs within a social environment, such that ER is influenced by the social context *and* that ER strategies contribute to how this social context is created. Whereas the social context effects on emotions and ER have received considerable attention (Gross and John, 2003), much less attention has been paid to the role of ER for the structuring of social relationships. Whereas quantity, quality, and changes of social networks have been theorized to be based on individuals’ behaviors and emotions (e.g., Crosier et al., 2012), no research to date has examined the role of cognitive ER strategies in shaping friendship and conflict network dynamics. To address these gaps, we deployed an advanced longitudinal SNA approach (Snijders et al., 2010) to examine a quintessential question of what is the role of individual differences in ER strategy use for social relationships. Specifically, we sought to characterize the role of ER strategies for the creation and maintenance of friendship and conflict networks, while controlling for confounding network processes.

Our results demonstrated that, after controlling for several alternative explanations, individuals endorsing similar levels of adaptive ER were more likely to keep their friendship and conflict relationships over time. We also found that new conflict relationships were more likely to form over time among those who differed from each other in adaptive ER levels. Finally, individuals who endorsed higher levels of maladaptive ER were less likely to maintain existing conflict relationships and more likely to create new conflict connections over time. These results advance the knowledge about the consequences of *intra-individual* ER strategies for *interindividual* processes of creation and maintenance of friendship and conflict networks. In addition, our findings also demonstrate how SNA can be used to expand the understanding of how ER strategies contribute to the dynamic existence of relationships, how ER has social consequences beyond the dyad, and how individual and group goals may shape ER decisions and their consequences. This study illustrates the conceptual and methodological benefits of SNA for charting the new directions in emotion science research by explicating how ER occurs within and shapes the social context.

### Similarity on Adaptive ER Promotes Friendship and Conflict Maintenance

Our finding that similarity, or homophily, in adaptive ER increased the likelihood that individuals would remain in existing friendships is in line with our hypotheses. Because adaptive ER reduces distress and promotes positive affect (Gross and John, 2003), employment of this strategy by both friends and ensuing positive affective climate in a friendship could increase reciprocal liking and perceived success of a friendship (Clark and Taraban, 1991) and motivate individuals to work harder to maintain the friendship (Van Kleef, 2009). Indeed, evidence suggests that strong affective bonds, coupled with shared values and goals, support relationship maintenance (Slotter and Gardner, 2011). Similar endorsement of adaptive ER strategies, whether high or low, may facilitate effective communication in relationships, which has been linked to relational stability (Butler and Randall, 2013). For instance, similarity in emotion suppression was linked to higher relationship quality in romantic dyads (Velotti et al., 2016).

That similarity in adaptive ER also increased the likelihood of continued participation in conflict-laden relationships was striking. Adaptive ER is associated with increased levels of *distress tolerance*, suggesting that individuals endorsing such ER strategies are able to withstand higher levels of negativity and distress (Van Eck et al., 2017). Increased distress tolerance may make it more palatable for a person with elevated adaptive ER levels to remain in a conflict-laden relationship over time. Presumably, maintaining an ongoing conflict-laden relationship would be distressing and challenging for individuals who are not able to reappraise and refocus as a part of their coping with everyday stressors and disagreements. Moreover, documenting that adaptive ER is associated with continued conflict-laden relationships may also underscore the adaptive function of this ER approach to prevent escalation when situation selection or modification strategies of ER are not possible or preferable options to regulate one’s emotions (Gross, 2015). Finally, research in industrial-organizational I/O psychology shows that adaptive ER strategies prevent a more frequent task-related conflict from escalating into a more damaging personal conflict (van den Berg et al., 2014). Unfortunately, due to participant burden, we did not distinguish between task and interpersonal conflicts and future work is needed to further test these conjectures.

These results highlight the central role played by the similarity in adaptive ER for continuation of *both* positive and negative relationships. This similar role of adaptive ER across diverse relationship types underscore that all categories of social relationships can place a toll on their constituent members and require ER. For example, using similar levels of adaptive ER as a friend can help the dyad members provide matching levels of emotional support to each other, while preventing burn-out and exhaustion, and increase the likelihood that the friendship is maintained over time. Conversely, using similar levels of adaptive ER in the context of a conflicted relationship can help protect dyad members from adverse effects of conflict through increased distress tolerance. These speculations suggest that distinct mechanisms may link similarity on adaptive ER strategies to perpetuation of positive and negative types of relationships. Future research is needed to better understand these mechanisms.

### Dissimilarity on Adaptive ER Leads to Conflict Creation

Findings also indicated that individuals who were not using the same levels of adaptive ER were more likely to create new conflict-laden relationships. This finding is in accord with co-regulation and shared regulation perspectives (Hadwin et al., 2018), suggesting that group dynamics are derived from individual *and* social dimensions of ER (Järvenoja and Järvelä, 2009). Conflict-laden relationships may be more likely to emerge among individuals who do not have the same toolkit of ER strategies for coping with stress. It could be that the effectiveness of “adaptive” ER strategies may be diminished when group members employ them at different levels.

### Maladaptive ER Strategies Lead to Cycling Through Conflict-Laden Relationships

Importantly, individuals who endorsed higher levels of maladaptive ER were more likely to terminate their existing conflict relationships and more likely to add new conflict relationships, suggesting that individuals who frequently use maladaptive ER strategies may be more apt for terminating ongoing conflicted ties. In accordance with the investment model advanced by Rusbult et al. (1998), individuals involved in negative relationships may lack the motivation or energy to engage in ER strategies to reduce their negative emotions or their desire to persist in the relationship. Similarly, experiencing high levels of relationship conflict likely leads to discontent and an avoidance of interactions that are perceived as detrimental to emotional health and/or a refusal to directly address relationship problems.

Relatedly, individuals who endorsed higher levels of maladaptive ER were more likely to create new conflicted connections over time. Perhaps, individuals who use maladaptive ER strategies may exaggerate the negative features of a manageable disagreement, thereby (Richards and Gross, 2000) and/or use punitive behavior when engaging with others (Buckholtz et al., 2008), both of which increase the likelihood that they will create new conflict-laden relationships. Lastly, individuals tend to shape their social networks to accommodate their specific emotional needs (English and Carstensen, 2014). Individuals use knowledge of their own ER capacities and those of individuals in their social networks to strategically address relational and emotional challenges. Relational conflict is inevitable (Deutsch et al., 2011), but the inability to resolve conflict can put friendships at risk. Individuals who consistently use maladaptive ER may view themselves as not having the competencies necessary to develop more positive relationships with individuals that they perceive as more skilled in terms of ER (Bonanno and Burton, 2013). Conversely, individuals who have difficulty accessing adaptive ER tend to avoid or otherwise escape situations that elicit negative emotions (Gratz and Roemer, 2004).

### Theoretical Implications

This study directly addressed how cognitive ER shapes the very existence and dynamics of social relationships, both positive and negative. Similarity in ER promotes the existence of relationships and recognizes that the social consequences of ER strategies expand beyond an actor and their partner to the group. Our novel contribution to the literature is that *similar levels* of adaptive ER strategies were more important in the context of social relationships—both friendships and conflict-laden—than the *overall level* of adaptive ER use. This pattern can be understood through Gross’s process model (Gross, 1998), which suggests that ER is a temporal phenomenon and that situation selection, situation modification, and attentional deployment often occur *before* cognitive change and response modulation can be activated (Gross, 2013). Commensurate with this model, the overall frequency of adaptive cognitive ER may not fully capture the overall ER efficacy of focal individuals, who also are likely to deploy a broader range of situation and attention-related ER approaches, which were not considered in this study and need to be systematically investigated. Nonetheless, our findings suggest that similarity in the frequency of adaptive ER may exert a stronger influence on relationships than individual levels of adaptive ER. These results also expand our understanding of the role of ER perception on relationship maintenance. For example, the perceived level of emotion suppression used in a romantic dyad was found to be related to relationship quality (Eldesouky et al., 2017).

The second important contribution of this research was that we explored how an individual’s use of ER prospectively influenced their interpersonal outcomes by observing how their ER use shaped their social network dynamics within an ecologically valid social context. Specifically, we demonstrated that *group* dynamics are affected by the cognitive ER an actor chooses. We build upon work that demonstrates the importance of ER to individual (Chervonsky and Hunt, 2019) and relational functioning in dyads (Butler and Randall, 2013) and establish that group-level outcomes of ER can and should be better understood. Our results suggest that network science provides potent tools for conceptualizing, measuring, and modeling social group dynamics.

### Limitations and Future Directions

The present results have several limitations that represent directions for future research. First, we were unable to distinguish between different types of conflict as potential moderators of associations between ER and these relational dynamics. Conflict can be interpersonal, ideological, and political, and individuals have different responses to these varying types of conflicts (Wainryb et al., 2001). Relatedly, the I/O literature distinguishes between task and interpersonal conflict (van den Berg et al., 2014), and ER strategy use may relate differently to task rather than personal conflict. Future research should examine the role of ER for different types of conflict among members of a social group. We also used self-report measures. Employing peer reports or naturalistic observational measures of these same constructs may yield different findings. Another limitation of this study stems from the notion that ER strategy use is guided by individual and group norms for how emotions should be expressed and regulated (i.e., emotional display rules). Endorsing similar emotional display rules may make it more likely that friendships will be maintained and less likely than conflict will occur (Matsumoto et al., 2008), especially if those norms are other- rather than self-oriented. Future studies would be prudent to add an emotional display rule assessment, which will enable better understanding of within-social network and cross-network comparisons. Still, caution is necessary in generalizing the results of this study with our sample and the type of network in which it was embedded to ER strategy use within other social groups. Relatedly, to manage participant burden not all types of emotion regulation could be included in the present study. Thus, future studies should be sure to incorporate additional conceptualizations of ER such as emotion avoidance.

Another limitation of our work is that we had no measure of relationship quality. Given that friendships are not equally beneficial, it is possible that those with similarly low endorsement of adaptive ER strategies tend to remain in friendships with toxic qualities or that friendships with toxic qualities are eliciting a similar need for regulation. In these situations, friendship maintenance may be detrimental to the focal individual *and* to the larger group. Future research should consider the qualitative aspects (e.g., intensity and quality) of friendship and conflict relationships as they relate to ER, both concurrently and over time. Finally, our measures of cognitive ER focused on *frequency* (i.e., how often a particular ER strategy is used; McRae et al., 2012). Hence, our results do not provide information about how other central aspects of ER, such as ability or self-efficacy (Gross, 2015) shape the dynamics of positive and negative relationships. This means that, although these findings help us understand the consequences of individual differences in ER frequency, more work needs to be done to understand how ER frequency and ability interact to impact the quality of social relationships.

## Conclusion

We investigated how cognitive ER strategies influence dynamics of social relationships over time within the context of an ecologically valid social network. Specifically, we found that similarity in adaptive ER strategy was associated with the maintenance of both friendships *and* conflict-laden relationships within the group. In addition, dissimilarity in adaptive ER endorsement was related to new conflict relationships formed over time. Thus, the social outcomes of regulation depend on more than the ER strategy chosen in isolation. Maladaptive ER strategy was associated with a perpetuating cycle of conflict-laden relationships in which new conflict ties were created and existing ones terminated. These results extend previous research by showing that maladaptive regulation relates to shorter duration of positively valenced relationships (Lopes et al., 2011), but adds to the literature by including how poor regulation impacts creating new negatively valenced relationships. Broadly then, the central finding of this study was that ER strategies are associated with changes in friendship and conflict connections among members of a large social group. This finding contributes to a more nuanced view of associations between ER strategy and relationship dynamics and moves the field toward a more ecologically valid understanding of how ER strategies impact social functioning and dynamics. Bridging network science theory and SNA methods into emotion science research can advance understanding of the consequences of individual- and group-level regulation of emotion for the nature and dynamics of social relationships.

## Data Availability Statement

Data is available with an appropriate IRB approval. Please direct inquiries to the corresponding authors.

## Ethics Statement

The studies involving human participants were reviewed and approved by the Arizona State University IRB. Written informed consent to participate in the study was obtained from all participants.

## Author Contributions

CR and OK conceptualized and designed the study, and performed the statistical analysis. OK collected and managed the data. CR wrote the first draft of the manuscript. All authors wrote and edited sections of the manuscript.

## Conflict of Interest

The authors declare that the research was conducted in the absence of any commercial or financial relationships that could be construed as a potential conflict of interest.

## Publisher’s Note

All claims expressed in this article are solely those of the authors and do not necessarily represent those of their affiliated organizations, or those of the publisher, the editors and the reviewers. Any product that may be evaluated in this article, or claim that may be made by its manufacturer, is not guaranteed or endorsed by the publisher.
