# Supplementary material for: The Role of Cognitive Emotion Regulation for Making and Keeping Friend and Conflict Networks
Source: Front Psychol. 2022 Apr 25;13:802629. doi: 10.3389/fpsyg.2022.802629 (PMC9082816; doi:10.3389/fpsyg.2022.802629)
Supplement: Supplementary file 1 [file Data_Sheet_1.PDF]

Supplementary Material

1.1 Supplementary Figures

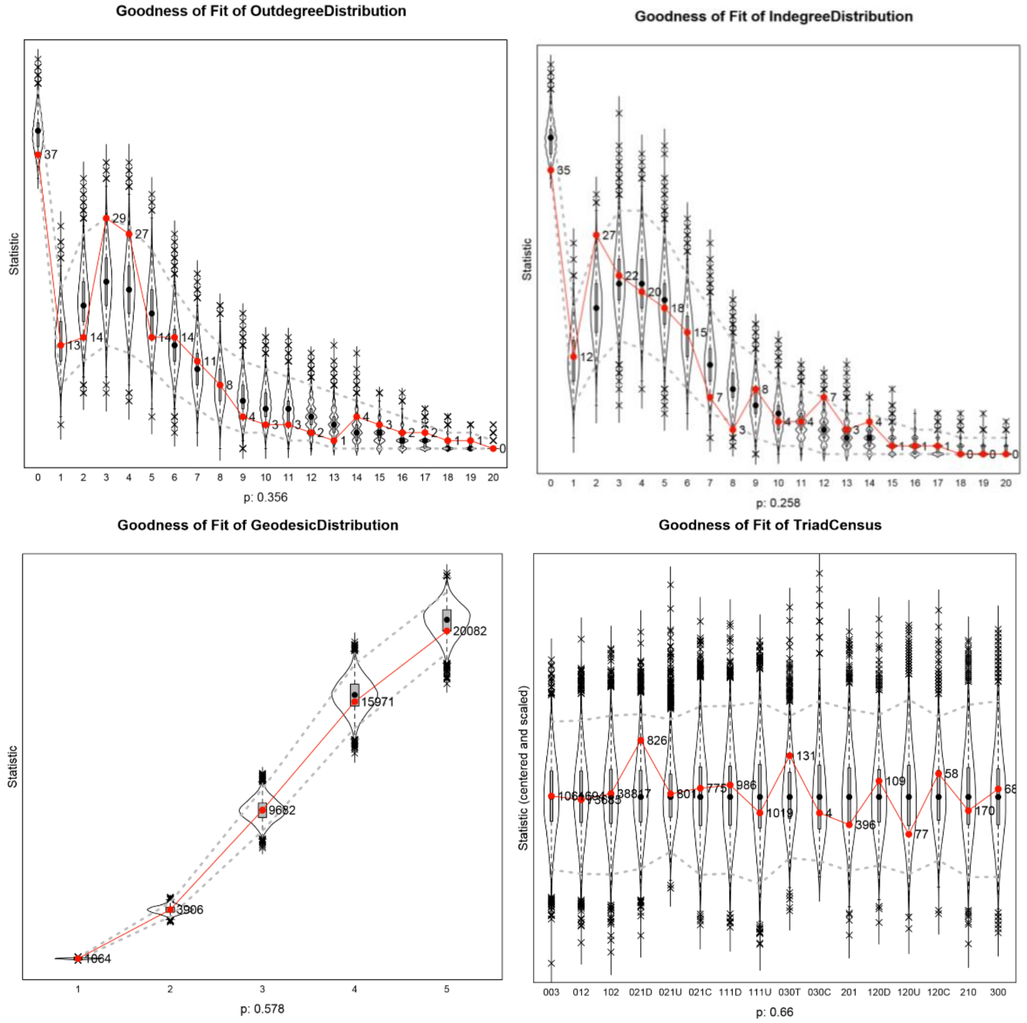

Supplementary Figure 1. Friendship network goodness of fit.

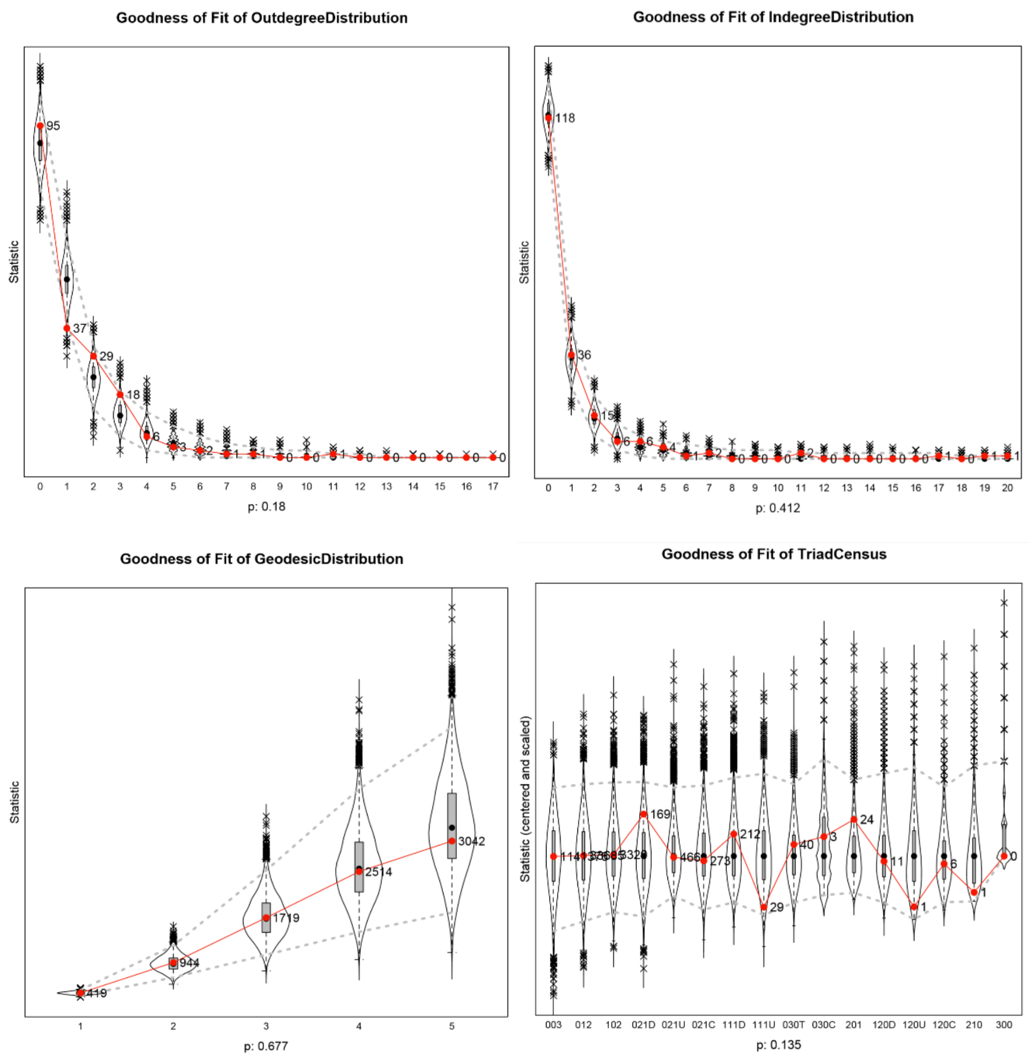

Supplementary Figure 2. Conflict network goodness of fit.
