# Supplementary material for: The Role of Cognitive Emotion Regulation for Making and Keeping Friend and Conflict Networks
Source: Front Psychol. 2022 Apr 25;13:802629. doi: 10.3389/fpsyg.2022.802629 (PMC9082816; doi:10.3389/fpsyg.2022.802629)
Supplement: Supplementary file 2 [file Data_Sheet_2.PDF]

Electronic Appendix 1

Graphic representation and interpretation of the SAOM parameters used in this study

Model Parameter	Figure	Interpretation (Rsiena shortname)
Effects of Covariates of Relationship Selection, Maintenance, and Creation Over Time		
Covariate ego / Activity	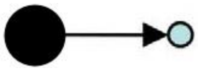	Actor *i* with higher values on a covariate (*v*) extends more outgoing ties {egoX}
Covariate alter /Popularity	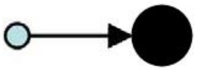	Actor *i* with higher values on a covariate (*v*) attracts more incoming ties {altX}
Covariate same/ Homophily	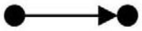	Actor i extends ties to alter *j* who has exactly the same values on a covariate (*v*), when *v* is a binary variable {sameX}
Covariate similarity/ Homophily	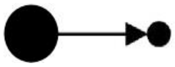	Actor *i* extends ties to alter *j* who is similar in values on a covariate (*v*), when *v* is a continuous variable {simX}
Network Structural Effects		
Outdegree	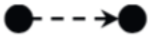	Actor *i* extending ties to alter *j* {density}
Reciprocity	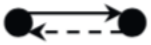	Actor i reciprocating ties to alter j {recip}
Transitive triplets	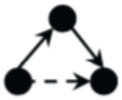	Actor *i* extending ties to alter *j* to whom he is indirectly tied (via actor *h*) {transTrip}
Transitive Reciprocated Triplets	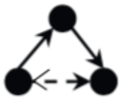	Actor *i* has a reciprocal tie to alter *j* to whom he is indirectly tied (via actor *h*) {transRecTrip}
3-cycles	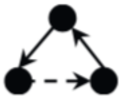	Actor *i* extending ties to alter *j* to whom he is indirectly tied (via actor *h*) {cycle3}
Transitive Ties	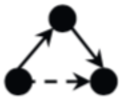	Actor *i* extending ties to alter *j* to whom he is directly and indirectly tied (via actor *h*) (one indirect tie suffices) {transTies}

Balance	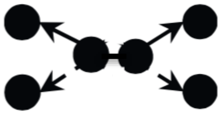	Similarity between the outgoing ties of actor *i* and the outgoing ties of the other actors *j* to whom i is tied {balance}
Number of actors at dist 2	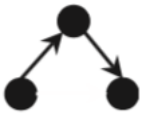	the number of actors to whom actor *i* is indirectly tied (through at least one intermediary, i.e., at sociometric distance 2) {nbrDist2}
Indegree – popularity (sqrt)	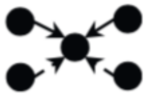	Actors with many incoming ties attract more incoming ties {inPopSqrt}
Outdegree – popularity (sqrt)	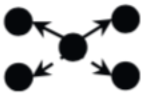	Actors with many outgoing ties extend more outgoing ties {outActSqrt}
Cross-Network Effects		
Conflict to agreement effect in friend network	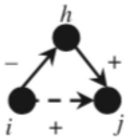	Conflict between *i* and *h* and friendship between *h* and *j* lead to friendship between *i* and *j* {to}
Friend to agreement effect in conflict network	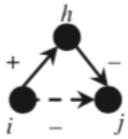	Friendship between *i* and *h* and conflict between *h* and *j* lead to conflict between *i* and *j* {to}
